# RRP Regulates Autophagy through the AMPK Pathway to Alleviate the Effect of Cell Senescence on Atherosclerosis

**DOI:** 10.1155/2023/9645789

**Published:** 2023-01-30

**Authors:** Dekun Liu, Yueyue Song, Tinging Song, Lin Lin, Lei Zhang, Qiong Yang, Xingchen Niu, Dan Liang, Jiufeng Yin, Wenqing Yang, Dan Zhang

**Affiliations:** ^1^Faculty of Traditional Chinese Medicine, Shandong University of Traditional Chinese Medicine, Jinan, China; ^2^Innovation Institute of Traditional Chinese Medicine, Shandong University of Traditional Chinese Medicine, Jinan, China; ^3^Faculty of Pharmacy, Shandong University of Traditional Chinese Medicine, Jinan, China; ^4^Shandong Province Cardiovascular Disease Chinese Medicine Precision Diagnosis Engineering Laboratory, Shandong University of Traditional Chinese Medicine, Jinan, China; ^5^Experimental Center, Shandong University of Traditional Chinese Medicine, Jinan, China; ^6^Key Laboratory of Traditional Chinese Medicine Classical Theory, Ministry of Education, Shandong University of Traditional Chinese Medicine, Jinan, China

## Abstract

Autophagy is closely associated with atherosclerosis and other cardiovascular diseases (CVD). Compound Danshen prescription is widely used as a clinical antiatherosclerotic drug. In our previous studies, we have shown that the combined active component, ginsenoside Rg1-notoginsenoside R1-protocatechualdehyde (RRP), can effectively alleviate endothelial dysfunction and reduce atherosclerotic plaques. However, the association between cellular senescence, caused by reduced autophagy, and atherosclerosis remains unclear. In this study, we investigated whether RRP can enhance autophagy and alleviate cell senescence through the AMPK pathway. Our results showed that RRP reduced the secretion of inflammatory factors in the serum of atherosclerotic mice, enhanced autophagy, and alleviated aortic aging in mice, thus reducing atherosclerotic plaques. In human aortic endothelial cells (HAECs), RRP effectively enhanced autophagy and inhibited senescence by activating the AMPK pathway. When AMPK*α* was silenced, the effect of RRP was inhibited, thus reversing its antiaging effect. Overall, our results show that RRP regulates autophagy through the AMPK pathway, thereby inhibiting cell senescence and alleviating the progression of atherosclerosis, suggesting that RRP may be a potential candidate drug for the treatment of atherosclerosis.

## 1. Introduction

Atherosclerosis can be caused by various factors, which lead to changes in the tissue microenvironment and initiate the secretion of proinflammatory factors, thereby promoting the development of atherosclerotic plaques [1, 2]. Autophagy is a major intracellular degradation system of dysfunctional organelles that is essential for cell renewal and homeostasis [3]. As autophagy is a mechanism that protects and kills stressed cells [4], defects in this system have been shown to promote endothelial cell senescence and apoptosis [5, 6]. Notably, atherosclerosis and cell senescence are self-promoting, leading to the development of many cardiovascular diseases (CVD) [7]. The progression of atherosclerosis can subsequently cause cell senescence, in which cells undergo unique phenotypic changes as a result of the action of a series of stimuli, causing cell cycle blockage. During senescence, cells secrete inflammatory cytokines, chemokines, growth factors, and proteases, which induce senescence-related secretory phenotypes (SASP) [8]. SASP can stimulate the immune system to clear senescent cells, but this process diminishes with aging, leading to the hypothesis that SASP affects nonsenescent neighboring cells through paracrine and autocrine mediators, thereby promoting atherosclerotic plaque progression and instability [7, 9, 10]. Previous studies have found that increased senescence of vascular endothelial cells leads to endothelial cell dysfunction, and senescent endothelial cells accumulate at sites of atherosclerosis. Consequently, the protection of endothelial cells from stress-induced premature senescence has become an area of focus in atherosclerosis therapy research.

AMPK functions as a receptor for cellular energy, affecting the metabolic pathways of protein, glycogen, and fatty acids. Additionally, it plays a protective role in the cardiovascular system by affecting the cell cycle and inhibiting cell proliferation in response to cardiovascular stimulation [11]. After AMPK activation, ATP supply is supplemented by autophagy and fatty acid oxidation [12]. AMPK is also a negative regulator of mTOR. AMPK phosphorylation inhibits mTOR expression, which induces autophagy in many different cell types [13]. Activating vascular wall cell autophagy can protect endothelial cells from damage and contribute to plaque stability [14], whereas inhibition of autophagy accelerates cell senescence, apoptosis, and necrosis, making plaques more susceptible to instability [15]. AMPK activation can reduce oxidative stress, promote autophagy, and play an anti-inflammatory role by delaying endothelial cell senescence and inhibiting the progression of atherosclerosis [16].

The Danshen prescription is mainly composed of ginseng, Panax notoginseng, and borneol. It can affect coronary artery contraction, inhibit platelet aggregation, and increase myocardial tolerance to hypoxia, making it an important antiatherosclerotic drug in traditional Chinese medicine [17]. In our previous study, we found that ginsenoside Rg1-notoginsenoside R1-protocatechualdehyde (RRP), the active component of Danshen, has antiatherosclerotic activity [17]. We have previously used RRP to antagonize endothelial cell injury and suggested that its protective function may be associated with the reduction of the inflammatory response caused by shear stress. Overall, the molecular mechanism through which RRP protects endothelial cells from senescence remains unclear. Based on previous studies, we speculated that the protective effect of RRP on endothelial cells may be mediated by the AMPK signaling pathway. Our results suggest that RRP can act through the AMPK signaling pathway to reduce oxidative stress, promote autophagy, and delay cell senescence. Therefore, the effects of this medicine have potential for application as a new atherosclerosis prevention and treatment strategy.

## 2. Materials and Methods

### 2.1. Animal Experiments

Eight-week-old male ApoE^−/−^ and C57BL/6 mice were purchased from Beijing Weitong Lihua Experimental Animal Technology Co., Ltd. (Beijing, China; animal certificate number SCXK (Jing)2021-0011). All animal experiments were conducted in accordance with the guidelines for the Care and Use of Experimental Animals (published by the National Institutes of Health) and were approved by the Advisory Committee on Animal Care and Research of Shandong University of Traditional Chinese Medicine. During the experiment, all mice were fed water and food freely at a temperature of 22 ± 2°C in a specific pathogen-free (SPF) laboratory under a 12 h/12 h light/dark cycle. After a week of adaptation, all mice were fed a high-fat diet. After 12 weeks of continuous high-fat diet, the ApoE^−/−^ mice were randomly divided into three groups: model group (receiving 0.9% sodium chloride; I.P.), Ros group (receiving 10 mg/kg^−1^ rosuvastatin; I.G.), and RRP group (receiving 10 mg/kg Rg1+10 mg/kg R1+14 mg/kg PCAD kg^−1^; I.P.). Fifteen C57BL/6J mice were used as the control group (receiving sodium chloride; I.P.). All animals were treated once daily for 8 weeks.

### 2.2. Histopathological Analysis

Aortic samples were stained with Oil Red O to evaluate the overall load and distribution of atherosclerosis. Excess tissue was peeled off and the entire aorta was opened longitudinally. Samples were fixed overnight with 4% PFA solution and then rinsed for 3 min with PBS before staining with 0.5% Oil Red O working solution for 10 min at 37°C. The entire aorta was then immersed in 70% ethanol and then rinsed with PBS. The samples were imaged using a stereo-fluorescence microscope (M205FA, LEICA). The degree of aortic atherosclerosis was evaluated based on the ratio of lesion area to aortic area.

Subsequently, the aortic sinus was fixed with a 4% PFA solution for 24 h and then treated with paraffin. The aortic sinus was cut into 5 *μ*m thick continuous paraffin sections. The aortic lipid plaque and elastin plaque areas were measured based on hematoxylin and eosin (H&E) and Movat staining (at room temperature). A pathological section scanner (HS6, SUNNY) was used to capture images.

### 2.3. Immunohistochemistry

Immunohistochemistry was used to detect the levels of *γ*-H2AX (1 : 250; cat. no. ab81299, Abcam) in aortic tissue. Briefly, the thoracic aorta was fixed overnight with 4% paraformaldehyde, embedded in paraffin, and cut into 5 *μ*m thick sections. Subsequently, the slices were incubated in an oven at 65°C for 2 h, dewaxed in xylene twice for 10 min, and dehydrated in 99%, 95%, and 75% ethanol for 5 min, at each concentration. The antigen was extracted using citric acid at boiling temperature for 20 min, hydrogen peroxide was then added, and the samples were incubated in a dark box at room temperature for 10 min to reduce endogenous peroxidase levels. The sections were then sealed with 5% fetal serum for 1 h, shaken, and incubated overnight with a specific primary antibody at 4°C. In the next day, the slices were warmed at room temperature for 30 min and then incubated with secondary antibodies at room temperature for 30 min before imaging using a fluorescence microscope (Vert.A1, ZEISS).

### 2.4. Enzyme-Linked Immunosorbent Assay (ELISA)

The concentrations of IL-1 *α* (cat. no. E-EL-M3059, Elabscience), IL-1 *β* (cat. no. E-MSEL-M0003, Elabscience), IL-6 (cat. no. E-MSEL-M0001, Elabscience), MMP-3 (cat. no. E-EL-M0626c, Elabscience), TNF-*α* (cat. no. E-MSEL-M0002, Elabscience), TGF- *β*1 (cat. no. E-EL-0162c, Elabscience), and MCP-1 (E-EL-M3001, Elabscience) in mice were determined using an ELISA kit. All tests were performed according to the manufacturer's instructions. Absorbance was measured at 450 nm using an automatic enzyme labeling instrument (Thermo Scientific, Multiskan MK3).

### 2.5. Cell Culture and Treatments

Human aortic endothelial cells (HAECs, cat. no. CP-H080, Procell) cultured in endothelial cell culture medium (ECM, cat. no. 1001, Sciencell) were supplemented with 5% fetal bovine serum, penicillin/streptomycin antibiotic solution, and endothelial cell growth supplement (ECGS). Endothelial cells were grown in a humid environment at 37°C with 5% carbon dioxide. The culture medium was changed every 48 h. The cells used in the experiment were from passages three to eight. To evaluate the effect of RRP on senescence, the cells were pretreated with RRP or 20 *μ*M metformin (Met, cat. no. S1741-1g, Beyotime) for 1 h. Subsequently, the HAECs were treated with 800 *μ*M H_2_O_2_ (cat. no. 88597-100ML-F, Sigma-Aldrich, Merck KGaA) for 8 h [10, 13]. In the cell experiments, all samples were tested three times using a safe and aseptic process.

### 2.6. Cell Proliferation Assay

According to the manufacturer's instructions, 100 *μ*L of culture medium was inoculated with the HAECs (5 × 10^3^ cells/well) in a 96-well plate. After adhesion, the cells were incubated in the ECM with or without H_2_O_2_. After the treatments, the cells were incubated at 37°C for 8 hours. Next, each well was supplemented with 10 *μ*L of CCK-8 solution (cat. no. MA0218-2, Meilunbio) solution and 90 *μ*L of ECM, and the plate was incubated in a carbon dioxide incubator at 37°C for 3 or 4 h. Absorbance at 450 nm was then determined using a full-wavelength enzyme labeling instrument (BioTek; Cytation 5).

### 2.7. Senescence-Associated *β*-Galactosidase Assay

To assess senescence, we used a senescence-associated *β*-galactosidase staining kit (cat. no. 9860, CST) according to the manufacturer's instructions. Briefly, after washing the HAECs several times with cold PBS, they were fixed for 15 min with 1 mL of fixing buffer at room temperature. After washing, the cells were mixed with 1 mL of staining solution and incubated overnight at 37°C. Images were obtained using a light microscope (TS2-S-SM, Nikon).

### 2.8. Immunofluorescence

After 48 h of treatment, HAECs were fixed for 15 min with 4% paraformaldehyde and washed with PBS three times. The cells were permeabilized for 10 min with 0.1% Triton X-100 and sealed with 5% BSA at room temperature for 1 h. After occlusion, cells were incubated with *γ*-H2AX (1 : 500; cat. no. 9718, CST) antibody overnight at 4°C, followed by incubation with Alexa Fluor 488 (cat. no. EF0008, SparkJade) secondary antibodies for 1 h at room temperature. The cells were stained with DAPI for 5 min and then observed under a fluorescence microscope (Vert.A1, ZEISS).

### 2.9. mRFP-GFP-LC3 Immunofluorescence

To detect mitochondrial autophagy, the cells were transfected with RFP-GFP-lc3-double fluorescent adenovirus (cat. no. GV540, GENE) and cultured in serum-free medium at 37°C for 8 h. The HEACs were then transferred and cultured in a complete medium for 48 h before observation by confocal microscopy (LSM880, ZEISS).

### 2.10. Cell Reactive Oxygen Species (ROS)

Intracellular ROS levels were measured using a reactive oxygen species detection kit (cat. no. S0033, Beyotime). For this, the HAECs cultured in 12-well plates were loaded with 10 mmol/L DCFH-DA and incubated for 25 min at 37°C. After washing step, the cells were observed under a ZEISS Vert.A1 fluorescence microscope.

### 2.11. mtROS and Mitochondrial Membrane Potential (MMP)

MitoSOX Red mitochondrial superoxide indicator (cat. no. M36008, Invitrogen) was used to treat the cells. The HAECs were mixed with 5 *μ*M of MitoSOX working solution and incubated for 10 min in the dark at 37°C. The cells were then washed gently three times with complete medium before observation of red fluorescence using a fluorescent microscope.

A JC-1 mitochondrial membrane potential detection kit (cat. no. C2006, Beyotime) was used to assess mitochondrial membrane potential. The cells were treated with JC-1 staining solution for 20 min in the dark and then washed twice with cold staining buffer. The stained cells were then observed under a fluorescence microscope (Vert.A1, ZEISS). Green fluorescence reflects the monomeric form of JC-1, and red fluorescence reflects the aggregated form of JC-1.

### 2.12. Analysis of Mitochondrial Respiration

Mitochondrial function was analyzed using an extracellular flux analyzer (Seahorse XFe96, Agilent). The HAECs were inoculated into ECM at a density of 8000 cells per well. After 12 h, the cells were treated with RRP for 2 h, supplemented with H_2_O_2_, and inoculated for another 8 h. The ECM was then replaced with the medium containing 25 mM glucose, and the cells were incubated in a carbon dioxide-free incubator at 37°C for 1 h. Oligomycin, FCCP, and antimycin A+rotenone were preloaded onto reagent ports A, B, and C, respectively, and oxygen consumption was calculated based on the slope of the change in concentration and time. Maximum respiration was calculated by subtracting the oxygen consumption rate when inhibited by rotenone+antimycin A from the oxygen consumption rate when inhibited by FCCP. ATP production was calculated by subtracting the oligomycin inhibitory oxygen consumption rate from the baseline oxygen consumption rate.

### 2.13. Transmission Electron Microscopy

After prefixing with 3% glutaraldehyde, the tissue was postfixed with 1% osmium tetroxide, dehydrated in a series of acetone solutions, infiltrated with Epox 812 for a longer period of time, and embedded. Ultrathin sections were cut using diamond knives and stained with uranyl acetate and lead citrate. The sections were examined using the JEM-1400-FLASH T.

### 2.14. Western Blot

For western blotting, the HAECs were cultured in 6-well plates. After the specified treatment, RIPA buffer, protease inhibitor, and phosphatase inhibitor were used to cleave HAECs or the mouse aorta. Protein concentrations were determined using the BCA method. Subsequently, the same amount of total protein was transferred to a PVDF membrane (90 min at 100 V) using an 8% PAGE Gel Super Fast Preparation Kit (cat. no. MA0381, Meilunbio). After transfer, the membrane was incubated at room temperature for 2 h in 5% skimmed milk dissolved at room temperature, and finally, the membrane was incubated with an antibody at 4°C overnight. The following primary antibodies were used: p16 (1 : 1000, cat. no. 18769, CST), beclin-1 (1 : 1000, cat. no. 3495, CST), NF-*κ*B p65 (1 : 1000, cat. no. 8242, CST), AMPK*α* (1 : 1000, cat. no. 2532, CST), p-AMPK*α* (1 : 1000, cat. no. 50081, CST), m-TOR (1 : 1000, cat. no. 2983, CST), p-mTOR (1 : 1000, cat. no. 5536, CST), p53 (1 : 1000, cat. no. 2524, CST), p21 (1 : 1000, cat. no. ab109199, Abcam), gamma-H2AX (1 : 5000, cat. no. ab81299, Abcam), eNOS (1 : 1000, cat. no. 32027, CST), p62 (1 : 1000, cat. no. ab91526, Abcam), lc3B (1 : 1000, cat. no. 43566, CST), and *β*-actin (1 : 1000, cat. no. 20536-1-AP, Proteintech). All membranes were washed with TBST three times and then incubated at room temperature for 1 h with secondary antibodies (goat anti-mouse IgG or goat anti-rabbit IgG) dissolved in TBST. Finally, the membranes were washed three times with TBST, and an ECL test kit was used for detection (cat. no. WBKLS0100; Millipore). Images were analyzed using ImageJ software and normalized using an internal control.

### 2.15. Total RNA Isolation and Quantitative Real-Time (qRT-PCR)

Total RNA was extracted from HAECs using the Simple P Total RNA Extraction Kit (BioFlux). Total RNA was quantified using NanoDrop One (Semel Fisher Science, USA), and 1 *μ*g of the RNA was reverse-transcribed into cDNA using the SPARKscript II RT Plus Kit (cat. no. AG0304, SparkJade). The qRT-PCR was performed using 2x SYBR Green qPCR Mix (cat. no. AH0104, SparkJade) and LightCycler 480 II (Roche). The sequences of the primers are listed in Table 1.

### 2.16. siRNA Transfection

HAECs were transfected with 100 nM siAMPK*α* according to the manufacturer's specifications. Briefly, the siRNA and transfection reagents were added to the siRNA transfection medium and incubated at room temperature for 30 min. The cells were then incubated with the siRNA transfection reagent for 6 h. The transfection mixture was subsequently removed, and cells were added to the complete medium and incubated for 48 h before the follow-up experiments. The expression of AMPK*α* was measured to evaluate the transfection efficiency.

### 2.17. Statistical Analysis

All results are expressed as mean ± standard deviation (SD). Student's *t*-test and one-way ANOVA were used to compare significant differences between more than two groups. Data are means of three biological repeats. All statistical analyses were carried out using GraphPad Prism 8.0 software. Differences were considered statistically significant at *p* < 0.05.

## 3. Results

### 3.1. RRP Attenuated the Vascular Senescence Induced by Atherosclerosis

**Figure 1:**
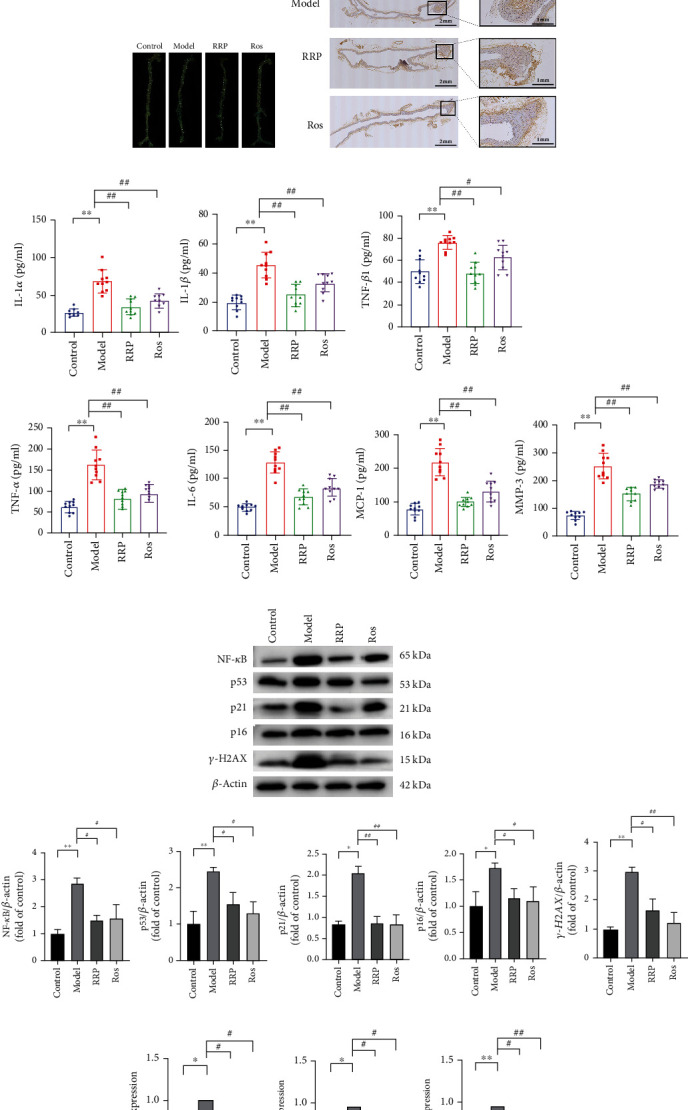
RRP reduces atherosclerosis-induced aging. (a) Representative Oil Red O staining of the entire aorta. (b) Representative images of H&E and Movat staining of aortic roots. (c) SA-*β*-gal staining to observe aortic vascular senescence in each group of mice. (d) Immunohistochemical assay to detect the levels of *γ*-H2AX in the blood vessels of each group of mice. (e) SASP levels in mice serum, including the levels of IL-1*α*, IL-1*β*, IL-6, MMP-3, TNF-*α*, TGF-*β*, and MCP-1. (f) Levels of senescence markers including NF-*κ*B, p53, p21, p16, and *γ*-H2AX in total vascular proteins of mice were detected by western blot. Data are shown as mean values ± SD per group and expressed as fold-over the control mean. (g) The amounts of mRNA senescence markers NF-*κ*B, p53, p21, and p16 in mouse vasculature were detected by qRT-PCR. Data are shown as mean values ± SD per group and expressed as fold-over the model mean (^∗^*p* < 0.05 and ^∗∗^*p* < 0.001 vs. control; ^#^*p* < 0.05 and ^##^*p* < 0.001 vs. model by one-way ANOVA test).

Atherosclerosis is an aging-related disease, and studies have shown that alleviating the effects of aging can effectively delay the progression of atherosclerosis [7, 18]. In this study, we evaluated the inhibitory effect of RRP on the progression of atherosclerosis in mice. We found that the Oil Red O-positive area in the model group was significantly larger than that in the control group, while RRP treatment significantly reduced the size of atherosclerotic plaques in the aorta (Figure 1(a)). Next we analyzed atherosclerotic plaques in the aortic root tissue sections and evaluated the severity of atherosclerosis based on H&E and Movat staining of the paraffin-embedded sections of the aorta. The aorta of ApoE^−/−^ mice showed severe atherosclerotic lesions with thicker plaques in the inner wall, outer wall, and intima, and a large number of cholesterol crystals were revealed by Movat staining (Figure 1(b)); however, these changes were greatly inhibited in ApoE^−/−^ mice administered with RRP.

Compared to untreated ApoE^−/−^ mice, the number of blue-stained areas in aortic SA *β*-gal staining was significantly reduced in the RRP group (Figure 1(c)). Immunohistochemical detection of *γ*-H2AX in the mouse aorta revealed that RRP decreased the levels of H2AX in ApoE^−/−^ mice (Figure 1(d)). We then detected senescence-related secretory phenotypes in the serum of these mice. Compared with the control group, the serum levels of TNF-*α*, IL-1 *β*, IL-6, IL-1 *α*, MMP-3, TGF-*β*1, and MCP-1 were significantly elevated in the model group (Figure 1(e)), whereas the overall level of SASP in the mice treated with RRP decreased, suggesting that RRP reduced the secretion of inflammatory factors and delay aging. We also found that RRP can reduce the levels of NF-*κ*B, which is an upstream activator of SASP, further demonstrating that RRP can reduce SASP expression (Figure 1(f)). Next we measured the expression levels of the aging markers p53, p21, p16, and *γ*-H2AX in the mouse aorta (Figures 1(f) and 1(g)). Compared with the model group, the expression of aging markers in the RRP group was lower than that in the model group. Taken together, these results show that RRP can delay vascular senescence in ApoE^−/−^ mice.

### 3.2. RRP Can Regulate the AMPK Pathway to Promote Autophagy in Atherosclerotic Mice

**Figure 2:**
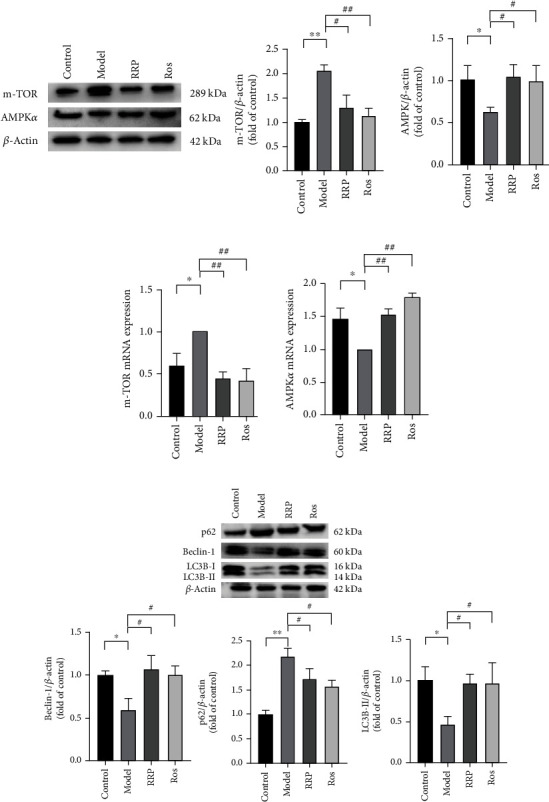
Activation of AMPK pathway by RRP promotes autophagy delaying vascular senescence. (a) Detection of AMPK*α* and m-TOR levels in total vascular proteins of mice by western blot. Data are shown as mean values ± SD per group and expressed as fold-over the control mean. (b) The amounts of AMPK*α* and m-TOR in the mRNA of mouse vasculature were detected by qRT-PCR in each group. Data are shown as mean values ± SD per group and expressed as fold-over the model mean. (c) Levels of autophagy-related proteins p62, beclin-1, and LC3B in total vascular proteins of mice. Data are shown as mean values ± SD of three animals per group and expressed as fold-over the control mean (^∗^*p* < 0.05 and ^∗∗^*p* < 0.001 vs. control; ^#^*p* < 0.05 and ^##^*p* < 0.001 vs. model by one-way ANOVA test).

Cell senescence is closely related to autophagy, which can reduce oxidative stress injury, eliminate damaged organelles, and protect cells [19, 20]. Next, we examined whether RRP could improve vascular aging in mice by regulating autophagy. Western blotting and qRT-PCR analysis revealed higher levels of AMPK*α* in mice treated with RRP than that in the model group and decreased levels of m-TOR, suggesting that RRP might inhibit m-TOR through the AMPK pathway, thereby promoting autophagy (Figures 2(a) and 2(b)). We also found that RRP increased the levels of beclin-1 and LC3B-II/I and decreased the levels of p62 protein, suggesting autophagy-associated damage (Figure 2(c)). These results demonstrated that RRP can mitigate aortic senescence in mice by enhancing autophagy through the regulation of the AMPK pathway.

### 3.3. RRP Can Reduce HAEC Senescence Induced by H_2_O_2_

**Figure 3:**
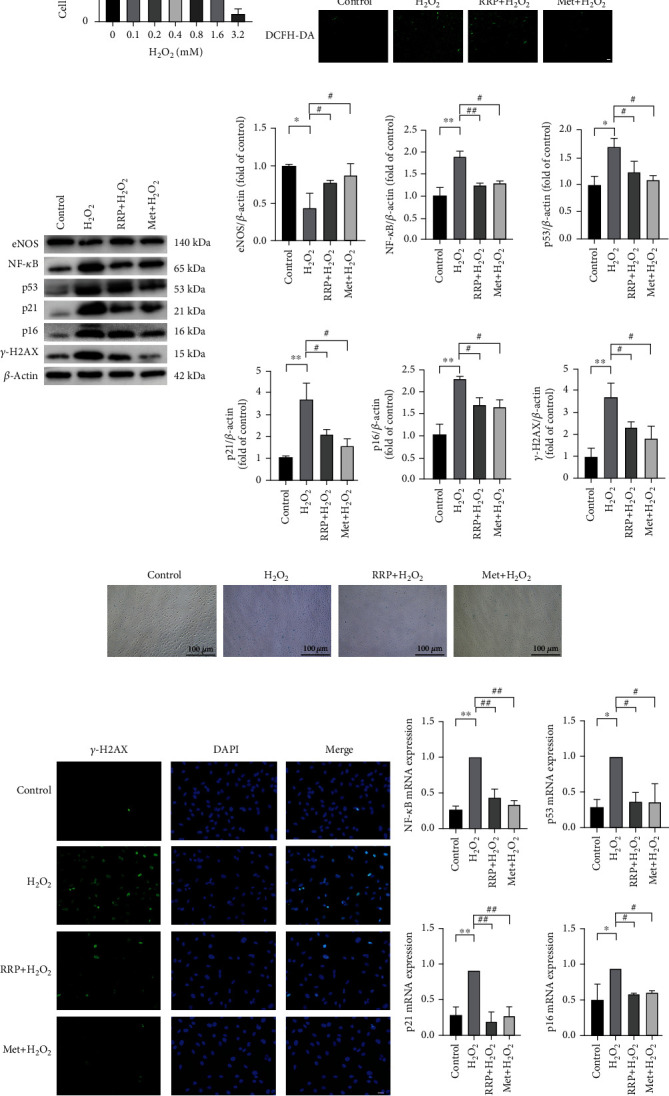
RRP attenuates H_2_O_2_-induced senescence in HAECs. (a) Different concentration gradients of H_2_O_2_ intervention in HAECs with cell viability were detected by the CCK-8 method. (b) DCFH-DA was used to detect the levels of ROS in each group of HAECs. Scale bar = 100 *μ*m. (c) Detection of senescence marker levels in the total protein of each group of HAECS by western blot. Data are shown as mean values ± SD per group and expressed as fold-over the control mean. (d) Representative staining images of SA-*β*-gal-positive cells after 2 h of early intervention with RRP or Met and 8 h of H_2_O_2_ intervention. (e) Immunofluorescence assay to detect *γ*-H2AX fluorescence levels in HAECs. Scale bar = 50 *μ*m. (f) Quantitative amounts of mRNA senescence markers in each group of HAECS were detected by qRT-PCR. Data are shown as mean ± SD per group and expressed as fold-over the H_2_O_2_ mean (^∗^*p* < 0.05 and ^∗∗^*p* < 0.001 vs. control; ^#^*p* < 0.05 and ^##^*p* < 0.001 vs. H_2_O_2_ by one-way ANOVA test).

Senescent endothelial cells are considered potential pathological factors in the development of vascular diseases [21, 22]. Therefore, to further study the effect of RRP on cell senescence, 800 *μ*M H_2_O_2_ was used to interfere with HAEC to induce oxidative stress damage and promote senescence (Figure 3(a)). First, we measured the level of oxidative stress damage after H_2_O_2_ intervention and used a DCFH-DA fluorescence probe to detect reactive oxygen species (ROS). We found that H_2_O_2_ treatment increased intracellular ROS levels (Figure 3(b)). The expression of the antioxidant nitric oxide synthase (eNOS) protein was decreased, and this effect was alleviated in the RRP group (Figure 3(c)). Thus, RRP intervention reduced the H_2_O_2_-induced oxidative stress in HAECs. Next, we used SA-*β*-gal staining to detect the degree of cell senescence (Figure 3(d)) and found that treatment with H_2_O_2_ significantly increased the number of SA-*β*-gal cells, whereas RRP pretreatment attenuated the H_2_O_2_-induced cell senescence. The levels of *γ*-H2AX were detected by immunofluorescence and western blot (Figures 3(c) and 3(e)), revealing that H_2_O_2_ increased the levels of *γ*-H2AX, whereas RRP reduced the levels of *γ*-H2AX. Additionally, RRP also decreased the levels of NF-*κ*B protein indicating that RRP could partially alleviate the DNA damage in HAECs caused by H_2_O_2_. We also observed increased protein and RNA levels of p53, P21, and P16, which are key markers of senescence in HAECs treated with H_2_O_2_. In contrast, after cotreatment with RRP, the amounts of p53, P21, and P16 were reduced (Figures 3(c) and 3(f)). These results indicated that RRP reduces oxidative stress and inhibits HAEC senescence.

### 3.4. RRP Regulates Autophagy in H_2_O_2_-Induced Senescence HAECs

**Figure 4:**
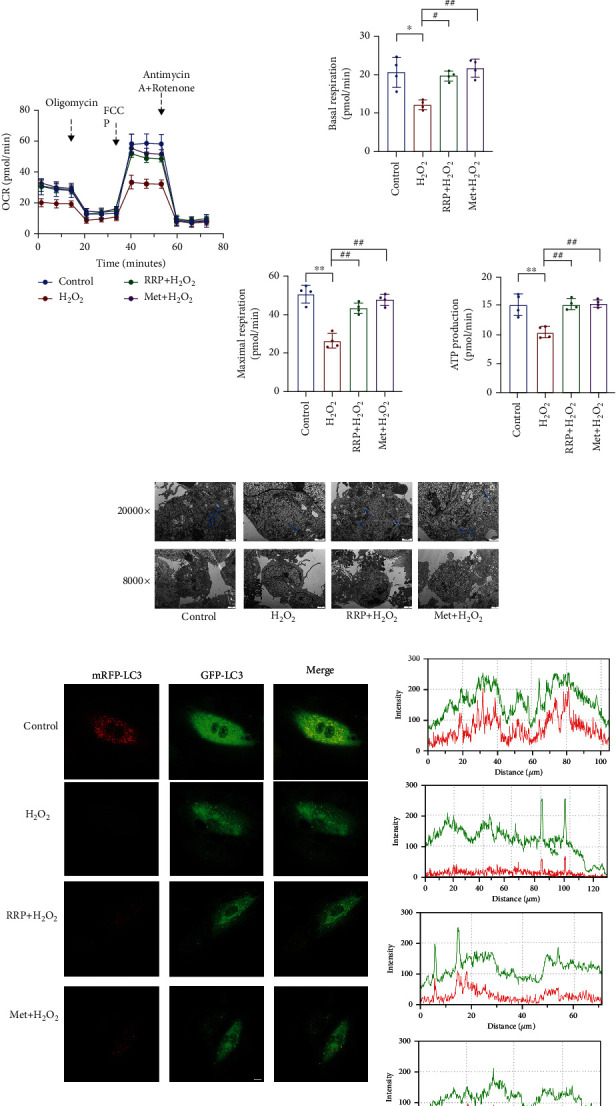
RRP protects the autophagic function of HAECs. (a) MitoSOX Red staining to detect mtROS levels in each group. Bar = 50 *μ*m. (b) Fluorescence microscopic observation of JC-1 probe to detect mitochondrial membrane potential. Scale bar = 100 *μ*m. Data are shown as mean values ± SD. (c) Representative OCR profile and maximal respiration, basal respiration, and ATP rate from three independent experiments. The metabolic inhibitors oligomycin (1.5 mM), FCCP (2 mM), and rotenone/antimycin A (0.5 mM) were injected into differently treated cells at the indicated time points. Data are shown as mean values ± SD. (d) Transmission electron microscopy to detect the change of autophagic vesicles in each group of HAECs. (e) Adenovirus mRFP-GFP LC3B transduction of HAECs, laser confocal microscopy to observe LC3B levels. Scale bar = 10 *μ*m. (f) Autophagy-associated protein levels in each group of HAECS as measured by western blot. Data are shown as mean values ± SD per group and expressed as fold-over the control mean (^∗^*p* < 0.05 and ^∗∗^*p* < 0.001 vs. control; ^#^*p* < 0.05 and ^##^*p* < 0.001 vs. H_2_O_2_ by one-way ANOVA test).

As a reactive oxygen species, H_2_O_2_ increases the level of oxidative stress in HAECs, which affects mitochondrial dysfunction and autophagy [10, 14, 23]. We first evaluated whether RRP could reduce the damage to HAEC mitochondria induced by H_2_O_2_ and used the MitoSOX fluorescence staining to detect mtROS (Figure 4(a)). The results showed that H_2_O_2_ significantly increased mtROS levels, suggesting mitochondrial damage, whereas RRP inhibited the increase in mtROS induced by H_2_O_2_. Next, we used the JC-1 probe to observe the MMP of HAECs. Compared with the control group, the ratio of red to green in the H_2_O_2_ group decreased significantly, indicating a decreased MMP level in the H_2_O_2_ group, while RRP increased the MMP level (Figure 4(b)). These results confirmed that RRP effectively alleviate the mitochondrial dysfunction in HAECs caused by H_2_O_2_. We also used a mitochondrial pressure detection kit to further investigate the protective effects of RRP on mitochondrial function and found that H_2_O_2_ significantly decreased the oxygen consumption rate (OCR) of HAECs, which was alleviated to varying degrees in the RRP and Met groups. Compared with the untreated cells, H_2_O_2_ treatment resulted in significantly reduced basal respiration and maximum respiration rates, whereas it was prevented by RRP pretreatment (Figure 4(c)). Moreover, the RRP pretreatment also restored the level of ATP, which was reduced following H_2_O_2_ treatment. These results suggest that RRP treatment protected the HAECs from oxidative stress-induced mitochondrial dysfunction.

Damage to mitochondrial function greatly affects the normal operation of autophagy, with reduced autophagic flux in senescent cells [24, 25]. Restoring autophagy partially alleviated cell senescence. Therefore, we further studied the effect of RRP on autophagy in HAECs induced by H_2_O_2_. After inducing cell senescence, we found that the numbers of HEAC autophagosomes associated with RRP pretreatment increased (Figure 4(d)). When autophagosomes are formed, the conversion of the cytoplasmic protein LC3B-I into LC3B-II and LC3B-II by enzymatic hydrolysis represents the initiation of autophagy. The expression of the autophagy marker LC3B was observed following adenovirus transduction of mRFP-GFPLC3 (Figure 4(e)). In H_2_O_2_-treated HAECs exhibiting senescence, autophagy marker LC3B and mitochondria were rarely colocalized (yellow dot), and this effect was alleviated in the RRP group. Consistent with this result, western blotting results showed that LC3B-II increased after RRP treatment compared with the H_2_O_2_ group, indicating that RRP increased the autophagy flux of HAECs. In addition, compared with the H_2_O_2_ group, the levels of the cell-associated autophagy protein beclin-1 increased and the levels of the autophagy substrate P62 protein decreased in the RRP group (Figure 4(f)). Together, these results suggest that RRP can mitigate mitochondrial damage and regulate autophagic flux, thereby reducing HEACs senescence.

### 3.5. RRP Attenuates H_2_O_2_-Stimulated HAEC Senescence by Activating AMPK Pathway

**Figure 5:**
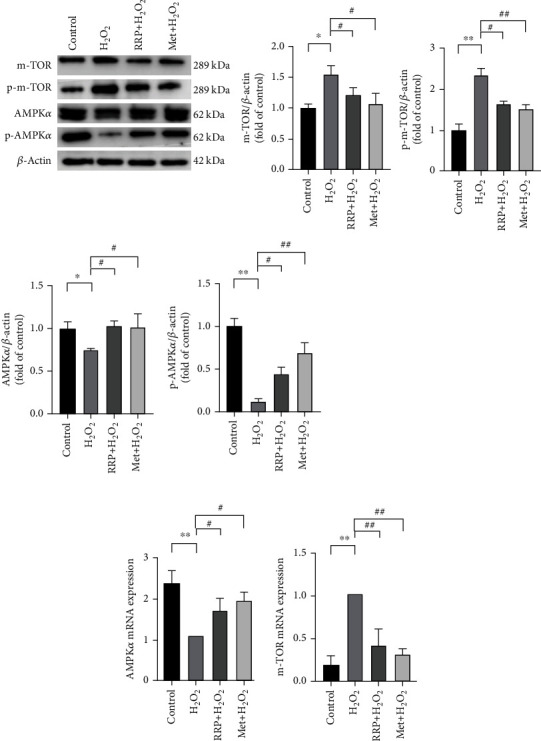
RRP delays aging by activating the AMPK pathway. (a) The levels of AMPK*α*, p-AMPK*α*, m-TOR, and p-m-TOR in the total protein of each HAECS group were detected by western blot. Data are shown as mean values ± SD per group and expressed as fold-over the control mean. (b) The amounts of AMPK*α* and m-TOR in mRNA of each HAECS group were detected by qRT-PCR. Data are shown as mean values ± SD per group and expressed as fold-over the H_2_O_2_ mean (^∗^*p* < 0.05 and ^∗∗^*p* < 0.001 vs. control; ^#^*p* < 0.05 and ^##^*p* < 0.001 vs. H_2_O_2_ by one-way ANOVA test).

AMPK is a late endosome/lysosomal resident protein that can be activated on the lysosomal membrane [14, 26], and AMPK pathway regulates autophagy and reduces cell senescence. To further clarify the effects of RRP on autophagy and senescence, we examined the expression of the AMPK pathway in HAECs [27]. As shown in Figure 5(a), the levels of AMPK*α* and p-AMPK*α* decreased to varying degrees in response to H_2_O_2_ treatment, and the downstream targets mTOR and p-mTOR were inversely activated. After treatment of senescent HAECs with RRP, the levels of AMPK*α* and p-AMPK*α* increased, and the levels of mTOR and p-mTOR decreased (Figures 5(a) and 5(b)). These results confirm that RRP affects the AMPK signaling pathway and regulates autophagy. In summary, RRP can alleviate H_2_O_2_-induced senescence in HAECs via the AMPK pathway.

### 3.6. siAMPK*α* Reversed the Effect of RRP on H_2_O_2_-Induced Senescence in HAECs

**Figure 6:**
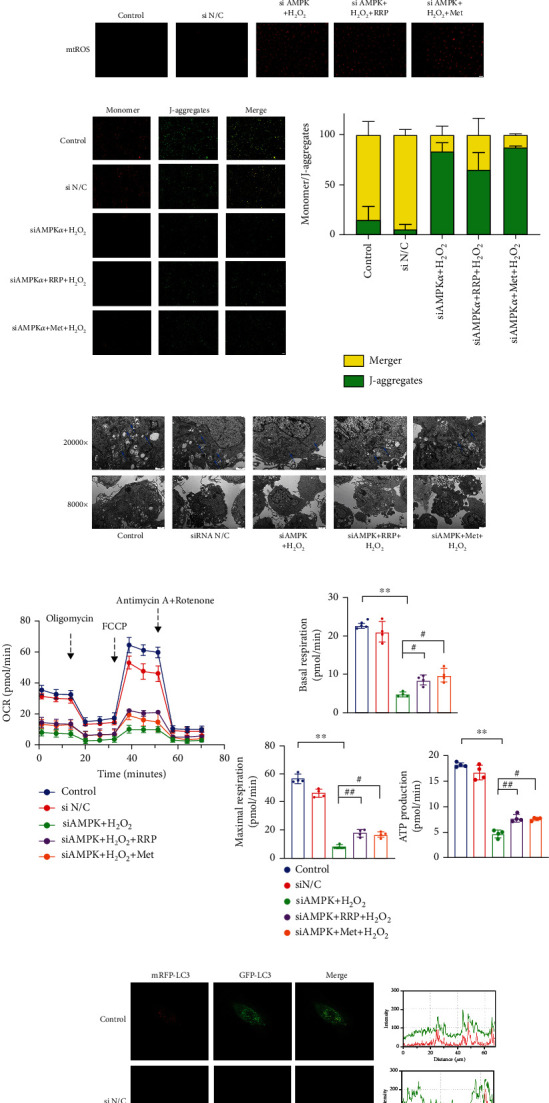
siAMPK*α* inhibited the protective effect of RRP on the autophagic function of HAECs. (a) siAMPK*α* intervention in HAECs for 8 h and AMPK*α* amounts in mRNA of HAECs after 2 d. (b) DCFH-DA detection of ROS levels in each group of HAECs after siAMPK*α* treatment. Scale bar = 100 *μ*m. (c) Levels of AMPK*α*, p-AMPK*α*, mTOR, p-mTOR, eNOS, p62, beclin-1, and LC3B in siAMPK*α*, H_2_O_2_, RRP, Met-treated, or untreated HAECs were detected by western blot. Data are shown as mean values ± SD per group and expressed as fold-over the control mean. (d) Detection of mtROS levels in each group by MitoSOX Red staining after siAMPK*α* treatment. Scale bar = 50 *μ*m. (e) Detection of mitochondrial membrane potential by JC-1 probe after siAMPK*α* treatment in HAECs. Scale bar = 100 *μ*m. Data are shown as mean values ± SD. (f) Transmission electron microscopy to detect the change of autophagic vesicles in each group of HAECs. (g) Levels of OCR profile and maximal respiration, basal respiration, and ATP rate in HAECs after siAMPK*α* treatment by transmission electron microscopy to observe the levels of autophagic vesicles in HAECs after siAMPK*α* treatment. Data are shown as mean values ± SD. (h) Laser confocal microscopy observation of adenovirus-transduced mRFP-GFP LC3B levels in HAECs after siAMPK*α* treatment. Scale bar = 10 *μ*m. (i) Detection of AMPK*α* and mTOR levels in siAMPK*α*-treated HAECs by qRT-PCR. Data are shown as mean values ± SD per group and expressed as fold-over the H_2_O_2_ mean (^∗^*p* < 0.05 and ^∗∗^*p* < 0.001 vs. control; ^#^*p* < 0.05 and ^##^*p* < 0.001 vs. siAMPK*α*+H_2_O_2_; ♢ = ns vs. siAMPK*α*+H_2_O_2_ by one-way ANOVA test).

**Figure 7:**
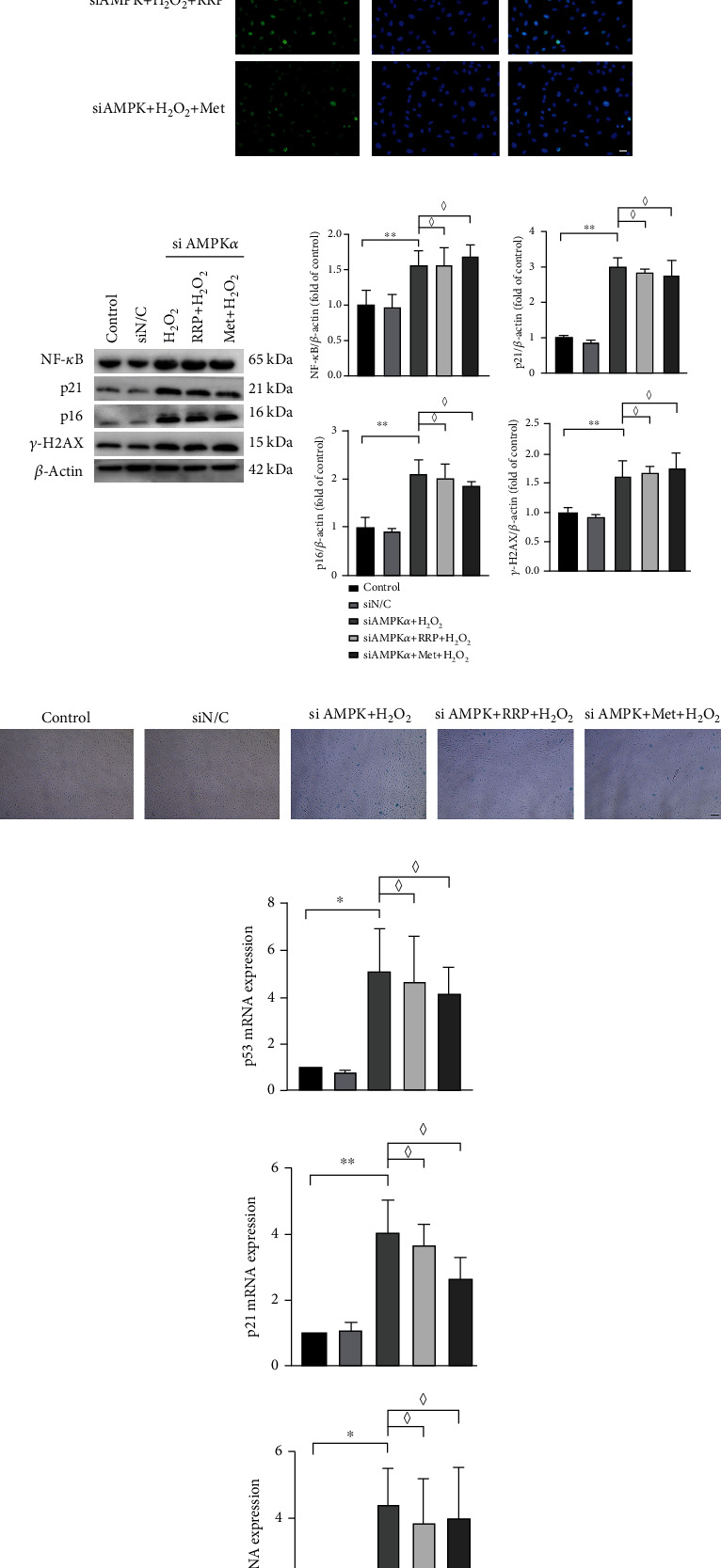
siAMPK*α* reversed the antisenescence effect of RRP. (a) Detection of *γ*-H2AX levels in HAECs after siAMPK*α* treatment by immunofluorescence assay. Scale bar = 50 *μ*m. (b) Detection of senescence marker levels in HAECs after siAMPK*α* treatment by western blot. Data are shown as mean values ± SD per group and expressed as fold-over the control mean. (c) Representative staining images of SA-*β*-gal-positive cells in HAECs after siAMPK*α* treatment. Scale bar = 100 *μ*m. (d) Detection of quantitative amounts of mRNA senescence markers in HAECs after siAMPK*α* treatment by qRT-PCR. Data are shown as mean values ± SD per group and expressed as fold-over the H_2_O_2_ mean (^∗^*p* < 0.05 and ^∗∗^*p* < 0.001 vs. control; ♢ = ns vs. siAMPK*α*+H_2_O_2_ by one-way ANOVA test).

AMPK is one of the most important proteins regulating cell energy metabolism, and deletion of AMPK leads to altered autophagy [21, 28]. To better understand the role of RRP in the AMPK pathway, we studied the effect of siAMPK*α* on RRP. For this, we transfected HAECs with siAMPK*α* to silence AMPK*α* (Figure 6(a)). After siAMPK*α* treatment, RRP did not effectively reduce the levels of ROS (Figure 6(b)), and the levels of eNOS protein did not change (Figure 6(c)), indicating that when AMPK*α* was silenced, RRP could not effectively reduce oxidative stress damage in HAECs. We further examined the effect of RRP on HAEC autophagy following AMPK*α* silencing. Our results showed that cotreatment with RRP and siAMPK*α* did not effectively change the levels of mtROS (Figure 6(d)), MMP (Figure 6(e)), autophagosomes (Figure 6(f)), OCR values (Figure 6(g)), and autophagy marker LC3B in HAECs (Figure 6(h)). Additionally, RRP did not restore the levels of the autophagy-related proteins beclin-1 and LC3B-II, with no significant changes in the levels of m-TOR, p-m-TOR, and p62 proteins (Figures 6(c) and 6(i)). These results suggest that silencing AMPK*α* inhibits the effect of RRP on autophagy in HAECs. We also measured the levels of *γ*-H2AX and NF-*κ*B after silencing AMPK*α* and found that the ameliorative effect of RRP on DNA damage was inhibited (Figures 7(a) and 7(b)). At the same time, the results of SA-*β*-Gal assays showed that although RRP reduced the number of blue-stained cells (Figure 7(c)), this effect was weaker than that in the nonsilent AMPK*α* group. The levels of senescence-related proteins P21 and P16 also did not decrease significantly (Figures 7(b) and 7(d)). Finally, AMPK*α* knockout inhibited cell senescence regulation by RRP. Overall, the results of this study showed that the protective effect of RRP on HAEC senescence induced by autophagy-mediated H_2_O_2_ was partly dependent on the AMPK signaling pathway.

## 4. Discussion

Aging is a major risk factor for many diseases including atherosclerosis [7, 29, 30]. Senescent endothelial cells have been found in atherosclerotic plaques in elderly patients, and endothelial cell senescence is considered a potential pathophysiological factor in the development of cardiovascular disease [21, 31, 32]. This study is the first to demonstrate that RRP can attenuate H_2_O_2_-induced cellular senescence in HAECs by inducing autophagy via the activation of the AMPK signaling pathway.

Autophagy is a self-protective mechanism that plays an important role in the aging process. Damaged organelles and proteins are engulfed by autophagosomes and eventually degraded, allowing cells to maintain normal functions [33, 34]. Several studies have confirmed that in aged mice, the ECS expresses lower levels of autophagy proteins than in young mice [35]. Based on a recent hypothesis, the alleviation of senescence by autophagy may be associated with SASP regulation, as the accumulation of senescent endothelial cells can release SASP inflammatory cytokines, thereby promoting the development of vascular diseases [36]. Since activation of autophagy has been shown to reduce inflammation [37], SASP in senescent ECs was inhibited by the modulation of autophagy. This is consistent with our findings that RRP treatment significantly reduced the expression of inflammatory factors and alleviated aortic vascular senescence in ApoE^−/−^ mice. Although further studies are needed to determine how defective autophagy promotes ECS senescence, p62 is associated with mTORC1 or NF-*κ*B. The interaction between p62 and NF-*κ*B can partially explain SASP transcription, defective autophagy, and senescent growth arrest [38]. It is known that p62 induces SASP production through TRAF6 polyubiquitination, which enables NF-*κ*B activation [39]. Furthermore, increased p62 levels in autophagy-inhibited cells inhibited proteasomal degradation, leading to accumulation of protein aggregates. Finally, p62 is part of the mTORC1 complex and is required for mediating amino acid sensing, which activates the mTOR pathway [40]. Therefore, p62 accumulation may lead to overactivation of these pathways and promote cellular senescence. In this study, we used H_2_O_2_ to induce oxidative stress in HAECs, thereby damaging the cells by increasing intracellular ROS production, which subsequently led to impaired autophagy and accelerated cellular senescence. We evaluated the effect of RRP treatment on the levels of ROS, mtROS, MMP, and OCR and found that RRP restored impaired mitochondrial function and thus helped to preserve autophagy. Consistent with this result, the autophagy-related markers LC3B-II and beclin-1 were significantly upregulated, the autophagic substrate p62 was downregulated, and there was an increase in autophagic vesicles compared to the model group, together suggesting that RRP enhanced cellular autophagy, thereby delaying senescence.

AMPK plays an important role in cellular and organismal energy metabolism as an energy sensor and regulator [15, 16]. AMPK is sensitive to changes in ATP depletion, with growing evidence showing that AMPK regulates lipid metabolism, inflammation, and angiogenesis in various animal models and cell types [41]. It cooperates with downstream molecules, including mTOR, a major regulatory pathway regulating autophagy in mammalian cells. Another study showed that decorin (DCN), a small leucine-rich proteoglycan, activates autophagy by activating AMPK and simultaneously inhibiting mTOR [42]. Pharmacological inhibition of mTOR by AMPK activators, such as rapamycin, significantly prolonged the lifespan of nematodes, Drosophila, and mice and showed AMPK-dependent inhibition in nematodes and mice [43–45], with significant results for SASP inhibition [46]. It is now generally accepted that the activation of AMPK attenuates cellular oxidative stress and inhibits m-TOR, thereby protecting cellular autophagy levels and exerting anti-inflammatory effects to delay cellular senescence [14, 44].

**Figure 8:**
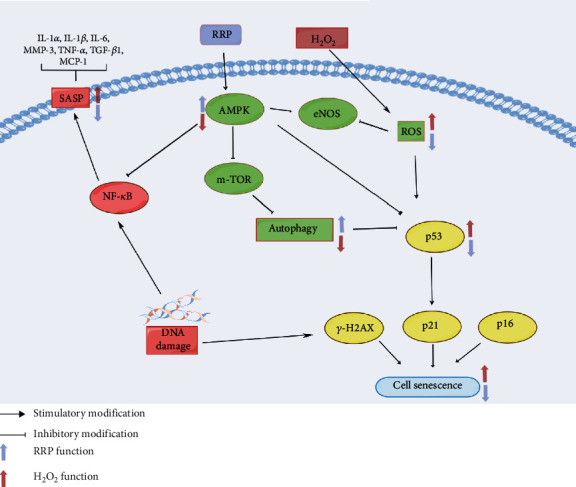
RRP plays a key role in the treatment of atherosclerosis by inducing autophagy and alleviating cellular senescence through the activation of the AMPK signaling pathway.

The regulation of the AMPK pathway by RRP has not been previously reported. We hypothesized that RRP would be able to activate the AMPK pathway and observed that early intervention of HAECs by RRP increased the levels of AMPK and p-AMPK and decreased the levels of downstream mTOR and p-m-TOR. Subsequently, we examined the levels of senescence markers SA-*β*-gal, p53, p21, p16, and *γ*-H2AX and found that RRP attenuated senescence in HAECs. Activation of autophagy by RRP was inhibited when AMPK*α* was silenced, whereas the antiaging effect of RRP was reversed. RRP attenuated ApoE^−/−^ mouse aortic SA-*β*-gal staining, reduced the secretion of senescence-related markers and SASP, and restored the levels of autophagy-related proteins. We further demonstrated that RRP regulates autophagy by activating the AMPK pathway (Figure 8).

In conclusion, our study using in vitro and in vivo models of atherosclerosis-induced cellular senescence demonstrated that RRP treatment produces a significant antiaging effect by inducing autophagy through the AMPK signaling pathway. This study provides new insights into the potential of RRP treatment and suggests that RRP may be a promising drug candidate for the prevention of endothelial cell senescence and atherosclerosis. However, this study had some limitations. First, aging is related to functions such as tissue repair, and therefore, careful evaluation of the dynamics of aging is required [7, 47]. Additionally, the association between autophagy and cellular senescence can vary owing to differences in the drugs, response time, cellular activity, and other therapeutic factors [7, 48]. Consequently, further studies are required to explore the detailed mechanisms underlying RRP-regulated autophagy. In conclusion, the results of this study indicate that the protective effect of RRP may be associated with autophagy, suggesting that RRP may have potential for application in the treatment of atherosclerosis.

## Figures and Tables

**Table 1:** Custom primer information.

Primer name	Sequence (5′ to 3′)
MUS-AMPK-91 R	TTCGGCAACCAAGAACGGTA
MUS-AMPK-91 F	GACCCCACTTCTCTTCGCAA
MUS-MTOR-193 F	GACCCCACTTCTCTTCGCAA
MUS-MTOR-193 R	CATAGTCAGGAGCCATCCGC
MUS-P53(TRP53)-80 F	GGCAACTATGGCTTCCACCT
MUS-P53(TRP53)-80 R	TTGAGGGGAGGAGAGTACGTG
MUS-P21(CDKN1A)-107 F	TTGTCGCTGTCTTGCACTCT
MUS-P21(CDKN1A)-107 R	TAGAAATCTGTCAGGCTGGTCT
MUS-P16(CDKN2A)-199 F	GCTCTTCTGCTCAACTACGGT
MUS-P16(CDKN2A)-199 R	CGATGTCTTGATGTCCCCGC
MUS-NFKB1-174 F	TGCCAAAGAAGGACACGACA
MUS-NFKB1-174 R	TGAGCATTGACTTCTGCCCC
MUS *Β*-ACTIN-154 F	GGCTGTATTCCCCTCCATCG
MUS *Β*-ACTIN-154 R	CCAGTTGGTAACAATGCCATGT
HOMO *Β*-ACTIN-163 F	CTCACCATGGATGATGATATCGC
HOMO *Β*-ACTIN-163 R	AGGAATCCTTCTGACCCATGC
HOMO-PRKAA1(AMPKAL)-151 F	GGAGCCTTGATGTGGTAGGAA
HOMO-PRKAA1(AMPKAL)-151 R	TCAAATAGCTCTCCTCCTGAGAC
HOMO-MTOR-82 F	AAGGTCTATTTGCCTCGCGT
HOMO-MTOR-82 R	TTGCCTTCTGCCTCTTATGGG
P53(TP53)-73 F	AGTCACAGCACATGACGGAG
P53(TP53)-73 R	GCCAGACCATCGCTATCTGA
HOMO-P21(CDKN1A)-112 F	TGTCTTGTACCCTTGTGCCT
HOMO-P21(CDKN1A)-112 R	TGGTAGAAATCTGTCATGCTCGTC
HOMO-P16(CDKN2A)-71 F	TCGCGATGTCGCACGGTA
HOMO-P16(CDKN2A)-71 R	CATCTATGCGGGCATGGTTACTG
HOMO-NFKB1 F	CCGTTGGGAATGGTGAGGTC
HOMO-NFKB1 R	CGCGTAGTCGAAAAGGGCAT

## Data Availability

The data used to support the findings of this study are included within the article.

## References

[1] Yancey P. G., Jerome W. G. (1998). Lysosomal sequestration of free and esterified cholesterol from oxidized low density lipoprotein in macrophages of different species. *Journal of Lipid Research*.

[2] Steinberg D., Parthasarathy S., Carew T. E., Khoo J. C., Witztum J. L. (1989). Beyond cholesterol. Modifications of low-density lipoprotein that increase its atherogenicity. *The New England Journal of Medicine*.

[3] Parzych K. R., Klionsky D. J. (2014). An overview of autophagy: morphology, mechanism, and regulation. *Redox Signal*.

[4] D'Arcy M. S. (2019). Cell death: a review of the major forms of apoptosis, necrosis and autophagy. *Cell Biology International*.

[5] Grootaert M. O. J., Roth L., Schrijvers D. M., De Meyer G. R. Y., Martinet W. (2018). Defective autophagy in atherosclerosis: to die or to senesce?. *Oxidative Medicine and Cellular Longevity*.

[6] Tian X., Yu C., Shi L. (2018). MicroRNA-199a-5p aggravates primary hypertension by damaging vascular endothelial cells through inhibition of autophagy and promotion of apoptosis. *Experimental and Therapeutic Medicine*.

[7] Machado-Oliveira G., Ramos C., Marques A. R. A., Vieira O. V. (2020). Cell senescence, multiple organelle dysfunction and atherosclerosis. *Cell*.

[8] Childs B. G., Li H., van Deursen J. M. (2018). Senescent cells: a therapeutic target for cardiovascular disease. *Journal of Clinical Investigation*.

[9] Childs B. G., Baker D. J., Wijshake T., Conover C. A., Campisi J., van Deursen J. M. (2016). Senescent intimal foam cells are deleterious at all stages of atherosclerosis. *Science*.

[10] Hernandez-Segura A., Nehme J., Demaria M. (2018). Hallmarks of cellular senescence. *Trends in Cell Biology*.

[11] Cantó C., Auwerx J. (2010). AMP-activated protein kinase and its downstream transcriptional pathways. *Cellular and Molecular Life Sciences*.

[12] Hardie D. G. (2011). AMP-activated protein kinase: an energy sensor that regulates all aspects of cell function. *Genes & Development*.

[13] Ou H., Liu C., Feng W., Xiao X., Tang S., Mo Z. (2018). Role of AMPK in atherosclerosis via autophagy regulation. *Science China. Life Sciences*.

[14] You G., Long X., Song F. (2020). Metformin activates the AMPK-mTOR pathway by modulating lncRNA TUG1 to induce autophagy and inhibit atherosclerosis. *Drug Design, Development and Therapy*.

[15] Jenkins A. J., Welsh P., Petrie J. R. (2018). Metformin, lipids and atherosclerosis prevention. *Current Opinion in Lipidology*.

[16] Vasamsetti S. B., Karnewar S., Kanugula A. K., Thatipalli A. R., Kumar J. M., Kotamraju S. (2015). Metformin inhibits monocyte-to-macrophage differentiation via AMPK-mediated inhibition of STAT3 activation: potential role in atherosclerosis. *Diabetes*.

[17] Zhang L., Li Y., Ma X. (2021). Ginsenoside Rg1-notoginsenoside R1-protocatechuic aldehyde reduces atherosclerosis and attenuates low-shear stress-induced vascular endothelial cell dysfunction. *Frontiers in Pharmacology*.

[18] Karnewar S., Neeli P. K., Panuganti D. (2018). Metformin regulates mitochondrial biogenesis and senescence through AMPK mediated H3K79 methylation: relevance in age-associated vascular dysfunction. *Biochimica et Biophysica Acta - Molecular Basis of Disease*.

[19] Wang X., Zhang J.-Q., Xiu C.-K., Yang J., Fang J.-Y., Lei Y. (2020). Ginseng-Sanqi-Chuanxiong (GSC) extracts ameliorate diabetes-induced endothelial cell senescence through regulating mitophagy via the AMPK pathway. *Oxidative Medicine and Cellular Longevity*.

[20] Li C., Lin L., Zhang L. (2021). Long noncoding RNA p21 enhances autophagy to alleviate endothelial progenitor cells damage and promote endothelial repair in hypertension through SESN2/AMPK/TSC2 pathway. *Pharmacological Research*.

[21] Sabbatinelli J., Prattichizzo F., Olivieri F., Procopio A. D., Rippo M. R., Giuliani A. (2019). Where metabolism meets senescence: focus on endothelial cells. *Frontiers in Physiology*.

[22] Xu S., Ilyas I., Little P. J. (2021). Endothelial dysfunction in atherosclerotic cardiovascular diseases and beyond: from mechanism to pharmacotherapies. *Pharmacological Reviews*.

[23] Uryga A. K., Bennett M. R. (2016). Ageing induced vascular smooth muscle cell senescence in atherosclerosis. *The Journal of Physiology*.

[24] van Deursen J. M. (2014). The role of senescent cells in ageing. *Nature*.

[25] Tyrrell D. J., Blin M. G., Song J. (2020). Age-associated mitochondrial dysfunction accelerates atherogenesis. *Circulation Research*.

[26] He P., Li Z., Xu F. (2020). AMPK activity contributes to G2 arrest and DNA damage decrease via p53/p21 pathways in oxidatively damaged mouse zygotes. *Frontiers in Cell and Developmental Biology*.

[27] Stojanović S. D., Fiedler J., Bauersachs J., Thum T., Sedding D. G. (2020). Senescence-induced inflammation: an important player and key therapeutic target in atherosclerosis. *European Heart Journal*.

[28] Zhan J. K., Wang Y. J., Li S. (2018). AMPK/TSC2/mTOR pathway regulates replicative senescence of human vascular smooth muscle cells. *Experimental and Therapeutic Medicine*.

[29] Williams J. W., Winkels H., Durant C. P., Zaitsev K., Ghosheh Y., Ley K. (2020). Single cell RNA sequencing in atherosclerosis research. *Circulation Research*.

[30] Kobiyama K., Ley K. (2018). Atherosclerosis. *Circulation Research*.

[31] Chan G. H., Chan E., Kwok C. T., Leung G. P. H., Lee S. M. Y., Seto S. W. (2022). The role of p53 in the alternation of vascular functions. *Frontiers in Pharmacology*.

[32] Lyu T. J., Zhang Z. X., Chen J., Liu Z. J. (2022). Ginsenoside Rg1 ameliorates apoptosis, senescence and oxidative stress in ox-LDL-induced vascular endothelial cells via the AMPK/SIRT3/p53 signaling pathway. *Experimental and Therapeutic Medicine*.

[33] Qiao L., Ma J., Zhang Z. (2021). Deficient chaperone-mediated autophagy promotes inflammation and atherosclerosis. *Circulation Research*.

[34] Hwang H. Y., Shim J. S., Kim D., Kwon H. J. (2021). Antidepressant drug sertraline modulates AMPK-MTOR signaling-mediated autophagy via targeting mitochondrial VDAC1 protein. *Autophagy*.

[35] LaRocca T. J., Henson G. D., Thorburn A., Sindler A. L., Pierce G. L., Seals D. R. (2012). Translational evidence that impaired autophagy contributes to arterial ageing. *The Journal of Physiology*.

[36] Grootaert M. O. J., Moulis M., Roth L. (2018). Vascular smooth muscle cell death, autophagy and senescence in atherosclerosis. *Cardiovascular Research*.

[37] Shi C. S., Shenderov K., Huang N. N. (2012). Activation of autophagy by inflammatory signals limits IL-1*β* production by targeting ubiquitinated inflammasomes for destruction. *Nature Immunology*.

[38] Sánchez-Martín P., Saito T., Komatsu M. (2019). p62/SQSTM1: ‘Jack of all trades’ in health and cancer. *The FEBS Journal*.

[39] Duran A., Linares J. F., Galvez A. S. (2008). The signaling adaptor p62 is an important NF-*κ*B mediator in tumorigenesis. *Cancer Cell*.

[40] Duran A., Amanchy R., Linares J. F. (2011). p62 is a key regulator of nutrient sensing in the mTORC1 pathway. *Molecular Cell*.

[41] Carling D. (2017). AMPK signalling in health and disease. *Current Opinion in Cell Biology*.

[42] Goyal A., Neill T., Owens R. T., Schaefer L., Iozzo R. V. (2014). Decorin activates AMPK, an energy sensor kinase, to induce autophagy in endothelial cells. *Matrix Biology*.

[43] Su Y., Wang T., Wu N. (2019). Alpha-ketoglutarate extends Drosophila lifespan by inhibiting mTOR and activating AMPK. *Aging*.

[44] Salminen A., Kaarniranta K. (2012). AMP-activated protein kinase (AMPK) controls the aging process via an integrated signaling network. *Ageing Research Reviews*.

[45] Martin-Montalvo A., Mercken E. M., Mitchell S. J. (2013). Metformin improves healthspan and lifespan in mice. *Nature communications*.

[46] Biomedicine L. M. (2013). A putative antiaging drug takes a step from mice to men. *Science*.

[47] Zhou X., Xu S. N., Yuan S. T. (2021). Multiple functions of autophagy in vascular calcification. *Cell & Bioscience*.

[48] Tyrrell D. J., Goldstein D. R. (2021). Ageing and atherosclerosis: vascular intrinsic and extrinsic factors and potential role of IL-6. *Nature Reviews Cardiology*.

